# Flexible metagenome analysis using the MGX framework

**DOI:** 10.1186/s40168-018-0460-1

**Published:** 2018-04-24

**Authors:** Sebastian Jaenicke, Stefan P. Albaum, Patrick Blumenkamp, Burkhard Linke, Jens Stoye, Alexander Goesmann

**Affiliations:** 1 Bioinformatics and Systems Biology, Justus-Liebig-University, Gießen, Germany; 2 Bioinformatics Resource Facility, Center for Biotechnology (CeBiTec), Bielefeld University, Bielefeld, Germany; 3 Genome Informatics, Faculty of Technology and Center for Biotechnology (CeBiTec), Bielefeld University, Bielefeld, Germany

**Keywords:** Metagenomics, Next-generation sequencing, Microbial community analysis

## Abstract

**Background:**

The characterization of microbial communities based on sequencing and analysis of their genetic information has become a popular approach also referred to as metagenomics; in particular, the recent advances in sequencing technologies have enabled researchers to study even the most complex communities.

Metagenome analysis, the assignment of sequences to taxonomic and functional entities, however, remains a tedious task: large amounts of data need to be processed. There are a number of approaches addressing particular aspects, but scientific questions are often too specific to be answered by a general-purpose method.

**Results:**

We present MGX, a flexible and extensible client/server-framework for the management and analysis of metagenomic datasets; MGX features a comprehensive set of adaptable workflows required for taxonomic and functional metagenome analysis, combined with an intuitive and easy-to-use graphical user interface offering customizable result visualizations. At the same time, MGX allows to include own data sources and devise custom analysis pipelines, thus enabling researchers to perform basic as well as highly specific analyses within a single application.

**Conclusions:**

With MGX, we provide a novel metagenome analysis platform giving researchers access to the most recent analysis tools. MGX covers taxonomic and functional metagenome analysis, statistical evaluation, and a wide range of visualizations easing data interpretation. Its default taxonomic classification pipeline provides equivalent or superior results in comparison to existing tools.

**Electronic supplementary material:**

The online version of this article (10.1186/s40168-018-0460-1) contains supplementary material, which is available to authorized users.

## Background

Metagenomics, the culture-free approach of sequencing and analysis of microbial communities, has emerged into a widely applied technique. Various studies have demonstrated its value not only in microbial ecology, but also in biotechnology and human diseases, deciphering the microbial community structure of natural [1], synthetic [2], or host-associated environments [3].

Identifying the composition of a microbial community and its functional capabilities are crucial steps towards determining its members’ roles within an ecosystem. In contemporary metagenomic studies, this is achieved by high-throughput sequencing and subsequent analysis utilizing reference databases to assign gene functions, enzyme classifications, or taxonomic origin to obtained DNA sequences. A variety of tools have been released employing different bioinformatics approaches, and all of them have contributed to advance the field; development of novel approaches and improvement of existing methods is still an active field of research, while initiatives like CAMI [4] (Critical Assessment of Metagenome Interpretation) strive to perform an independent evaluation of tool performance. Current sequencing technologies offer the opportunity to generate millions of DNA sequences within short time and at reasonable cost [5], making data analysis the new limiting factor, with costs for bioinformatics exceeding by far the expenses required for lab work and sequencing reagents.

Access to bioinformatics resources is limited, though metagenome analysis is a computationally demanding task [6], and established resources rely on public funding to offer their services. Recently, the well-known CAMERA portal [7] had to announce that it no longer accepts submissions due to lack of funding. The web-based IMG/M [8] and MG-RAST [9] platforms as well as the EBI metagenomics portal [10] continue to offer their services, serving as valuable resources to the community. A different approach is followed by CloVR [11], which is provided as a virtual machine image, and to a certain degree by CyVerse [12], a web-based infrastructure for data analysis that also provides a limited range of applications for metagenome analysis. However, these applications typically provide only one distinct set of analyses or a predefined pipeline, which might not be sufficient to investigate all questions arising from an underlying hypothesis. The discovery of novel biotechnologically relevant enzymes, for example, is difficult to achieve using existing solutions, as highly specific databases are often required to obtain the desired resolution [13]. In addition, the majority of recent algorithms are provided as command-line tools, and their use requires the provisioning of an appropriate compute infrastructure, and familiarity with programming if several tools need to be combined into an automated analysis pipeline.

At this point, workflow systems like for example Galaxy [14] or Conveyor [15] pose an interesting alternative to custom programming: Mainly specific to a certain application domain, they provide data processing capabilities in the form of small tasks; more complex analysis workflows are then devised by connecting these tasks into a pipeline or directed graph. Programming knowledge is not required, as a graphical user interface is usually provided to implement an analysis pipeline. The resulting workflow definition can be published along with its results, allowing for easy reproducibility of methods as well as serving as a self-documenting description. Furthermore, the building blocks of a workflow can easily be exchanged once an improved method becomes available: BLAST [16], for example, has been one of the most popular tools for database searches, despite its rather large computational overhead. Recently developed alternatives like GHOSTX [17] or DIAMOND [18] offer considerable acceleration compared to the original BLAST algorithm while retaining similar sensitivity. Employing a workflow engine, these alternatives can effortlessly be introduced as replacements for the BLAST program, an inevitable requirement to keep pace with the continuously growing output of next-generation sequencing machines.

However, adequate compute resources are still required, and workflow engines typically lack in both data management capabilities as well as support for appropriate visualizations, making them unsuitable as a sole means for metagenome analysis, unless they are used as part of a larger software platform.

## Implementation

### MGX overview

**Fig. 1:**
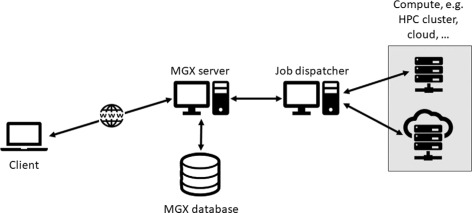
MGX system overview. MGX is a client/server framework, where each client connects to one (or several) MGX server instances; sequence data and corresponding metadata are stored on the server. A job dispatcher prioritizes and schedules analysis workflows to compute resources such as high-performance computing (HPC) clusters or a compute cloud

MGX is a framework for the analysis of metagenome data obtained by high-throughput sequencing. MGX is implemented as a client/server solution (Fig. 1) based on the Java programming language, which ensures maximum portability across a variety of commonly used operating systems such as MS Windows, Mac OS X, or Linux. The MGX GUI client can be easily downloaded and installed. By connecting to one of the public MGX server instances, users can analyze their data in an efficient and user-friendly manner without the need to establish and maintain a local compute infrastructure. In the following sections, we provide further details on the technical implementation of the whole platform, while the features of the graphical user interface are illustrated in the “Results and discussion” section.

### Server

During operation, the MGX client connects to one or several MGX application servers, which provide storage and compute resources for all hosted projects, managing project access and resource assignment. An MGX server instance consists of a dedicated J2EE-based application server (Oracle GlassFish) with RDBMS-provided relational (PostgreSQL) as well as flat-file storage systems. For efficient and fast access, sequence data is stored using an indexed and encoded format. The server also features dedicated storage resources allocated to each project, allowing users to upload own data to be included into analysis pipelines, e.g., own sequence collections, databases, or unpublished reference genomes. An associated job dispatcher takes care of prioritizing and scheduling analysis tasks, which might be scheduled for local execution, a DRMAA-compliant compute cluster, or cloud-based compute resources provided by the server operators. Access to MGX projects is managed by GPMS (General Project Management System), a system implementing Single Sign-On (SSO) developed at Bielefeld University. GPMS provides authentication and role-based access control: users may be granted administrative, standard or guest access, limiting allowed operations from full to read-only access. All features of the MGX framework are exposed via a REST-based interface, and communication between client and server is mandatorily encrypted, ensuring confidentiality of access credentials as well as unpublished data. All data transfers rely on Google’s Protocol Buffers format, which provides space-efficient encoding as well as fast serialization/deserialization.

For programmatic access, a MGX library is provided to remotely access and control a MGX server, thus allowing to automate routine analysis based on MGX.

### Scalability

A single MGX client is able to connect to several servers in parallel, and results are aggregated across project and server boundaries. Thus, MGX is easily scalable as more server instances are deployed, and researchers may even choose to operate their own MGX server with dedicated compute resources. At the same time, they are still able to access classification results for metagenomes residing on other servers, and comparative analyses can be performed without the need to re-execute classification pipelines.

### Workflow-based analysis

All analysis algorithms used within MGX are implemented as workflows based on the Conveyor workflow engine [15], which encapsulates common processing steps (list processing, filtering) as well as external bioinformatics tools (BLAST, HMMer) as simple nodes; pipelines are created by connecting nodes to build a directed graph representing the steps of an analysis. The Conveyor Designer (available from https://www.uni-giessen.de/fbz/fb08/Inst/bioinformatik/software/Conveyor/conveyor-designer) is a user-friendly GUI that enables researchers to implement custom analysis workflows, which can subsequently be uploaded into MGX. Conveyor already provides a wide set of bioinformatics tools and is constantly extended, with interfaces to other workflow engines being planned. A dedicated plugin serves as an interface between Conveyor and MGX, thus allowing sequence retrieval from and storage of analysis results in MGX; the plugin also provides the appropriate means to access files uploaded by the user, thus making it possible to incorporate custom data, e.g., sequence databases maintained within a research group, into an analysis pipeline. Additional plugins were implemented to support the most recent tools used for metagenome analysis, such as Centrifuge, Kraken, or GHOSTX. Finally, the well-known Mothur and QIIME suites have recently been integrated into Conveyor, extending the applicability of MGX from metagenomes to the analysis of 16S rRNA amplicon data, as well.

### Statistics

Several different statistical methods were integrated into the framework using the R statistical environment [19]. All statistical calculations are executed on the MGX server, which internally offloads them to a Rserve [20] instance. Most prominently, rarefaction, PCA, PCoA, hierarchical clustering, and M/A plots allow researchers to critically assess/compare results and determine major influential factors or to validate whether their data suffices to support a hypothesis or if additional sequencing should be conducted. Well-known biodiversity indices (Chao1, ACE, Shannon, Simpson) can be determined and used to relate own sequence data to other metagenomes obtained from similar environments.

### MGX repository

All predefined analysis pipelines are hosted in a central public repository with typically preset but customizable parameters, and each MGX server periodically updates its local copy of workflow definitions. MGX users may either choose to import a predefined pipeline into their project or provide a custom method implemented as a Conveyor workflow. Upon publication of a metagenome, we recommend custom pipelines to be published and integrated into the central MGX repository with all associated data for the benefit of all MGX users and to allow easy reproducibility of results.

In addition, each server provides published and annotated reference genomes, which can be used as targets for fragment recruitments or reference alignment (“mapping”) of metagenome sequences.

### Data model

Within MGX, each sequence data set is complemented by its metadata. In its data model (Additional file 1: Figure S1), MGX groups metadata into habitat, sample, DNA extract, and sequencing run entities, thus allowing to collect information about a metagenome’s origin together with sampling procedures and other treatments that might have an influence on analysis results.

Individual analysis results are modeled as observations associating a subregion of a DNA sequence with a certain attribute. Support for user-supplied workflows requires a highly generic representation of attributes, as actual characteristics are not necessarily known beforehand. Thus, attributes are stored in conjunction with a so-called attribute type, which specifies structure (flat, hierarchical) and domain (continuous or discrete values) of the referenced attribute, while the attribute itself provides only the actual value. Thereby, pipelines inherently define the type of results they are generating, and the MGX framework can automatically derive valid operations or determine for example appropriate visualization types for different kinds of results. At the same time, since results are retained on individual sequence level, MGX enables users to search for individual terms or export sequences based on arbitrary annotation data.

Finally, the data model stores precomputed versions of attribute distributions; accompanied with stored procedures, this approach enables fast access to summarized data for, for example, plots (almost completely) independent from a metagenome size.

### GUI client

The MGX client is a graphical user interface implemented based on the NetBeans Platform, a modular framework allowing for easy addition of new components. The designated application programming interface (API) facilitates future enhancements such as the development of new features or additional visualizations, which can be provided as NetBeans modules (NBM) or OSGi bundles and loaded into the application.

Since all computationally expensive analysis tasks are scheduled and executed on the server side, only little resources are required to run the MGX client application itself.

### Generation of benchmark datasets

To demonstrate the flexibility of the MGX platform, we implemented a combined taxonomic classification workflow and compared its results to those of several established standalone tools, which were selected based on recency and reported frequency of use according to literature: Kraken [21], Kaiju [22], Centrifuge [23], and MetaPhlAn 2 [24]. All tools were run with their respective default settings, with databases generated in May 2017, and classification performance was assessed at the genus level. For this, two artificial benchmark datasets were created using the Mason read simulator with a read length of 100 bp, an Illumina-specific error profile, and a mismatch probability of 3% (–seq-technology illumina –illumina-prob-mismatch 0.03).

In the first scenario (organism present in database), a reference database of complete archaeal and bacterial genomes restricted to one genome per species was created based on NCBI RefSeq. From this set of 2672 finished genomes, 5000 reads were obtained for each genome and the resulting artificial metagenome (13,360,000 reads) containing only fragments of known taxonomic origin was subsequently analyzed.

The second experiment (clade exclusion, organism not present in database) was performed to evaluate the classification performance based on sequences not contained in the database of each tool. For this, we compiled an additional synthetic metagenome based on NCBI GenBank, including only genomes where the annotated genus was present, but the species not present in NCBI RefSeq. From this set of 2376 genomes, an artificial metagenome comprising 11,880,000 reads was obtained as described above.

### Future development

MGX already offers a wide range of pipelines for both taxonomic as well as functional analysis (Additional file 1: Table S1) of metagenomes, which were chosen based on previous in-house studies or requested by cooperation partners for ongoing projects. Newly released software packages and relevant databases are provided as Conveyor plugins as soon as possible and offered to the MGX community in the form of predefined tools after passing internal quality control and evaluation. For the future, we also plan to create virtual machine images or Docker containers with the relevant software components and bioinformatics tools and databases to allow an easy setup of additional server instances.

## Results and discussion

### MGX—a versatile solution for metagenomics

We introduce MGX, a novel and flexible open-source framework for the analysis of metagenomic datasets. Implemented as a client/server solution, MGX supports the whole range of fundamental analysis types required for metagenome interpretation, including quality control, taxonomic profiling, functional analysis, and antimicrobial resistance screening, as well as descriptive statistics, multi-sample comparisons, and mapping of sequences to reference genomes.

MGX employs the Conveyor [15] workflow engine for all analysis pipelines and, in addition, also exposes it to users of the framework. Thus, researchers are able to devise completely new analysis types without any programming knowledge, and new workflows can be easily uploaded and executed on MGX-provided infrastructure. Also, the performance of tool combinations often exceeds that of individual tools, making it desirable to combine more than one program into a workflow to improve the quality of results.

Currently, MGX provides an extensive selection of over 30 predefined analysis pipelines commonly used for metagenome analysis, including, among others, sophisticated tools like Kraken [21] and Kaiju [22] as well as BLAST- and HMMer [25]-based assignments to KEGG pathways, Pfam [26], and TIGRFAM [27] protein families or COG [28] categories (see Additional file 1: Table S1). While all predefined pipelines already provide reasonable default settings, MGX allows users to adapt all parameters based on their own needs.

Flexibility of the MGX framework is achieved by its unique modeling of results, where attributes are stored in conjunction with additional information indicating the results’ properties, i.e., flat or hierarchical structure and numeric or discrete value types. Based on this result model, the framework supports arbitrary result types required for future novel analysis workflows or user-provided pipelines with custom output types.

### GUI client features

**Fig. 2:**
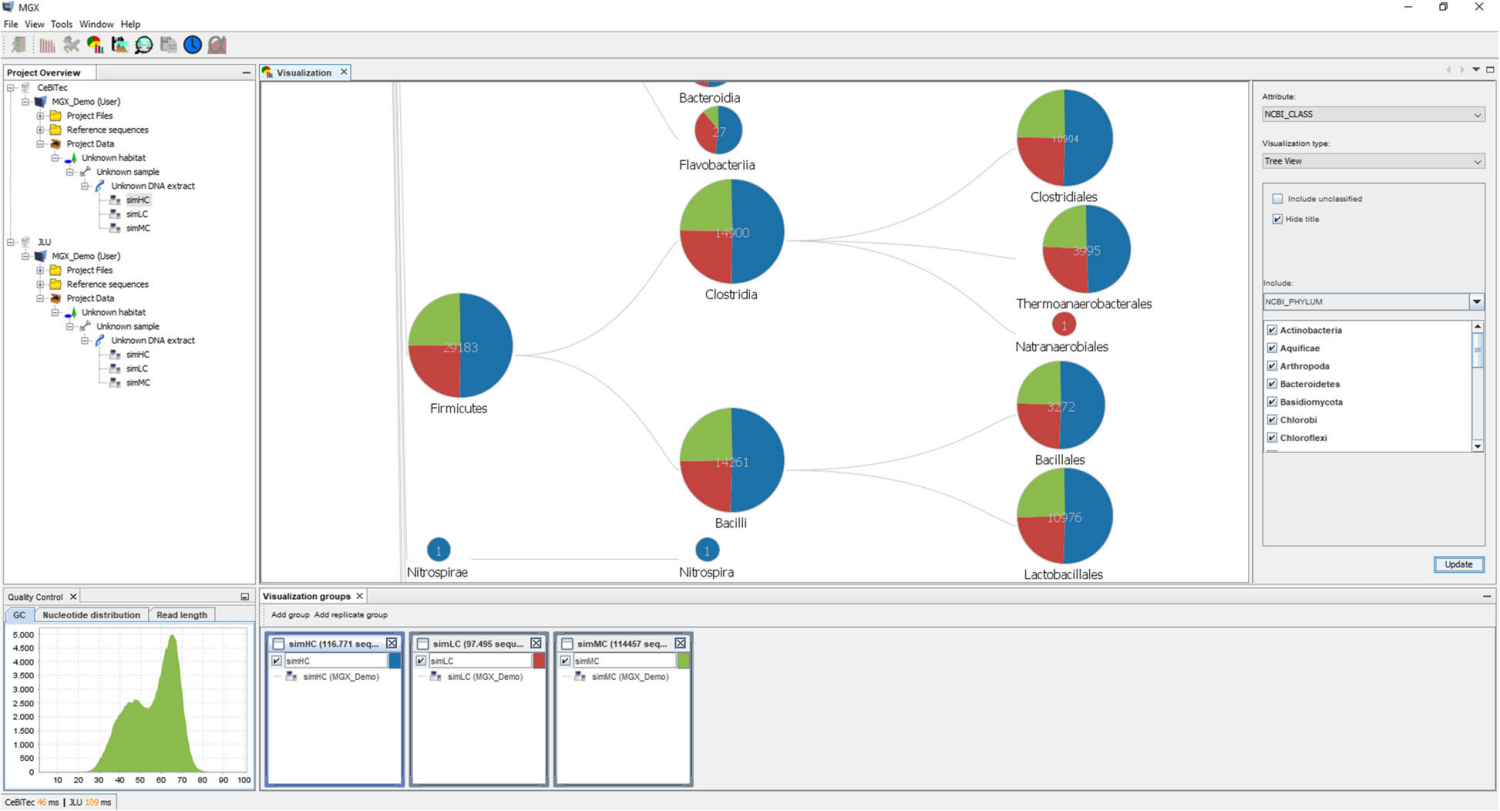
The MGX application client. Shown are the project explorer window (top left), quality control reports for the currently selected sequencing run (bottom left), and a hierarchical tree chart (center) displaying three groups, which are defined at the bottom. Different customization and filtering options are available for each chart (right)

**Fig. 3:**
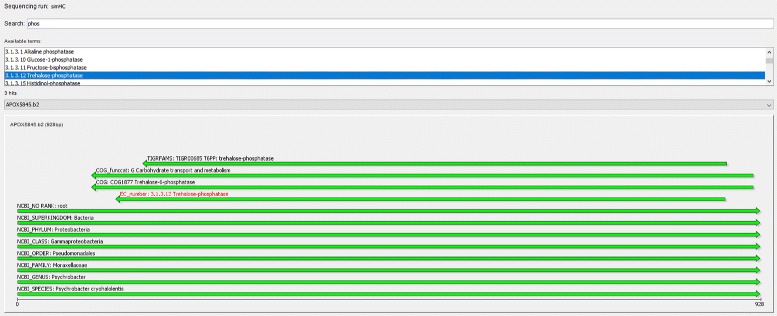
Single sequence resolution. With MGX, analysis results can be inspected down to individual sequence level, allowing the comparison between different annotation strategies as well as providing additional contextual information. Here, a sequence assigned to *Psychrobacter cryohalolentis* carries a trehalose-phosphatase fragment, which is independently supported by three different analysis methods (TIGRFAMS, COG, and an EC number)

The MGX client (Fig. 2) is an interactive application implemented in Java and available for all major operating systems. The graphical user interface allows to connect to multiple servers in parallel and assists the user in all common tasks, featuring convenient wizard-driven acquisition and validation of metadata (Additional file 1: Figure S2). After data upload, researchers can inspect quality reports for their metagenome datasets and are afterwards able to select one or several analysis pipelines, review and adapt existing parameters, and finally schedule the analysis jobs for execution. Upon completion, results can be retrieved and evaluated. The client features a rich set of different visualization modules, enabling researchers to interactively explore analysis results for their metagenome datasets, generate high-quality charts, or export results to for example Microsoft Excel. Based on the abstract modeling of results, the client application is able to automatically determine appropriate visualization types for different kinds of analysis results: Taxonomic assignments may be represented as hierarchical trees or as a bar chart showing only one taxonomic level; continuous data such as GC content or read length distribution will be displayed as a line or area chart, and metabolic functions can be shown as a table or mapped onto an appropriately colored KEGG pathway, just to give some examples. In the same manner, suitable postprocessing operations such as normalization or filtering are identified and offered to the user. For all visualizations, MGX allows users to freely define groups, thus allowing to combine and compare datasets across project or server boundaries. Various statistical methods are provided to investigate community complexity and coverage, as well as to identify determining factors in comparison between several metagenomes: rarefaction analysis allows researchers to estimate whether the amount of sequence data suffices to draw valid conclusions, biodiversity indices provide intrinsic measurements of community complexity, and several methods such as PCA, PCoA, M/A plots, or clustering can be utilized to interpret data in a comparative approach. All analysis results are stored on the individual sequence level; thus, MGX allows to export subsets based on user-given criteria, e.g., all metagenomic sequences assigned to a certain taxon or function; also, this enables researchers to trace results down to the individual sequence level (Fig. 3). Reference mappings (Additional file 1: Figure S3), the alignment of metagenome sequences to reference genomes of known origin, are another noteworthy feature and allow the creation of fragment recruitment plots (Additional file 1: Figure S4) based on public as well as user-supplied reference genomes.

### Flexible tool combination contributes to improved accuracy

The flexibility provided by MGX allows to implement sophisticated analysis pipelines based on multiple tools, resulting in improved overall performance. We demonstrate the advantages of this flexibility based on the default taxonomic classification workflow currently used by MGX. Inferring the taxonomic composition of a microbial community is one of the key aspects in metagenome analysis, and several standalone tools are available to accomplish this task. Here, the MGX framework relies on an advanced taxonomic classification pipeline which is based on a combination of the established Kraken [21] tool enhanced by an additional *lowest-common-ancestor* step using DIAMOND [18] and the RefSeq protein database [29]. This approach was chosen considering the high throughput of Kraken and the sensitivity offered by both approaches. Based on two artificial benchmark datasets, we evaluated the ability of our pipeline to classify reads of previously known as well as unknown taxonomic origin in comparison to established standalone tools like Kraken, Centrifuge [23], Kaiju [22], and MetaPhlAn 2 [24] (see Table 1).

**Table 1:** Taxonomic classification performance on genus level for benchmark datasets

	Kraken	Kaiju	Centrifuge	MetaPhlAn 2	MGX
RefSeq					
True positive	12,059,412	9,329,288	12,611,380	414,943	12,566,362
False positive	18,748	185,899	53,092	7,171	20,698
False negative	1,281,840	3,844,813	695,528	12,937,886	772,940
Sensitivity	0.9039	0.7082	*0.9477*	0.0311	0.9421
Precision	*0.9984*	0.9805	0.9958	0.9830	*0.9984*
Accuracy	0.9027	0.6983	*0.9440*	0.0311	0.9406
F1 score	0.9488	0.8224	*0.9712*	0.0602	0.9694
GenBank					
True positive	1,851,436	2,592,655	2,175,122	92,383	3,976,270
False positive	398,899	1,230,445	864,989	10,378	734,389
False negative	9,629,665	8,56,900	8,839,889	11,777,239	7,169,341
Sensitivity	0.1613	0.2435	0.1975	0.0078	*0.3568*
Precision	0.8227	0.6782	0.7155	*0.8990*	0.8441
Accuracy	0.1558	0.2182	0.1831	0.0078	*0.3347*
F1 score	0.2697	0.3583	0.3095	0.0154	*0.5015*

All tools achieve high precision on the RefSeq-derived metagenome, as the source organisms are already included in the relevant classification databases. For the GenBank-based metagenome containing only species not present in the tools’ databases, MetaPhlAn 2 offers high precision but only a very low sensitivity (0.78%), followed by the MGX-provided default pipeline, which ranks highest in sensitivity and accuracy as well as F1 score. Numbers in italics denote best results

In a first experiment, we evaluated the classification performance using simulated reads generated from the NCBI RefSeq genomes database; as NCBI RefSeq is also the origin of sequences used to initially create the classification databases for Kraken, Centrifuge, and Kaiju, all tools demonstrate a very high precision in excess of 98%, with Kraken and MGX showing the highest overall precision (both 99.84%) followed by Centrifuge (99.58%), MetaPhlAn 2 (98.30%), and Kaiju (98.05%).

For the second experiment, we created an artificial metagenome based on NCBI GenBank, explicitly selecting genomes where the species was not present in the classification databases of the individual tools (clade exclusion). In this experiment, MetaPhlAn 2 achieved the highest precision (89.90%) of all tools, but with a very low sensitivity of only 0.78% due to the fact that MetaPhlAn 2 is relying on a small set of marker genes. The MGX classification pipeline not only showed the second highest precision (88.41%), but also the highest sensitivity (35.68%), accuracy (33.47%), and F1 score (50.15%) of all evaluated tools. While all standalone tools already provide valuable results and correctly assigned a large number of sequences, the performance of the taxonomic analysis pipeline as implemented in MGX is either equivalent to or exceeding comparable tools. However, the majority of sequences remained unclassified by all of the tools, showing there is still a lot of room for future improvement in metagenomic sequence analysis.

### Comparison to existing platforms

Currently, several applications are available to users adressing the task of computational metagenome analysis, most notably MG-RAST, IMG/M, and the EBI metagenomics portal. Also, web-based infrastructures such as CyVerse provide some basic capabilities to process metagenome data, and virtual machines like CloVR enable metagenome processing on local or cloud-based resources (Table 2).

**Table 2:** Comparison of applications for metagenome analysis

	MGX	MG-RAST	IMG/M	EBI Metagenomics	CloVR	CyVerse
Quality control	x	x	x	x	–	x
Taxonomic/functional profiling	x	x	x	x	x	x
Assembly support	–	(x)^a^	(x)^a, b^	(x)^a^	–	x
Charts/visualizations	x	x	x	x	x	–
Custom pipelines	x	–	–	–	–	x
User-provided databases	x	–	–	–	–	x
Fragment recruitment	x	–	–	–	–	–
Free of charge	x	x	x	x	(−)^c^	x

^a^Supports analysis of preassembled data

^b^Submission restricted to assembled data

^c^No cost to run standalone virtual machine, but typical metagenome sizes will require additional compute resources on Amazon EC2

MG-RAST, IMG/M, and the EBI metagenomics website all provide a predefined analysis pipeline offering quality control and data analysis combined with either static or dynamic visualizations, but none of them allows users to benefit from more modern tools like Kraken or Centrifuge, implement own pipelines, or include custom databases. While IMG/M only accepts submissions of pre-assembled data for metagenomes sequenced outside of the JGI [30], MG-RAST and the EBI metagenomics portal are available without such restrictions.

The CloVR-Metagenomics protocol only provides very limited functionality in the form of a simple BLAST-based pipeline used for taxonomic and functional assignment with no preceding quality control; in addition, a single CloVR virtual machine does not provide sufficient compute power for the analysis of typical metagenome sizes in a reasonable time frame, and while CloVR allows to optionally distribute analysis jobs to cloud-based compute resources on Amazon EC2, this inflicts additional costs on the user.

Apart from MGX, the CyVerse infrastructure [12] so far remains the only environment offering at least some of the more recent tools for metagenome analysis and the ability to upload own reference databases; however, no complete analysis workflows are provided, and the user has to select and execute individual tools within their Discovery Environment. Also, the system does not provide any suitable visualizations, thus making it a more appropriate choice for advanced users and data analysts.

## Conclusions

With MGX, we provide a flexible platform for the analysis and interpretation of metagenome data. Its key strength is the unique combination of its abstract result model and workflow-driven analysis integrated into a scalable client/server framework. This combination facilitates the fast adoption of newly released tools, giving researchers access to the most recent algorithms, while at the same time providing them with a convenient user interface. For the first time, users gain the option of devising and executing own pipelines within a metagenomics platform, as MGX offers the inclusion of custom-tailored analysis methods; not only are researchers able to implement their own approach, but they can also provide custom resources such as sequence databases. Complemented with a wide range of predefined pipelines, MGX enables researchers to address both fundamental as well as highly specific questions relevant to their study aim. Until now, these types of analyses required custom programming or at least familiarity with the use of command line programs in addition to exhaustive compute resources such as supercomputers or large high-performance compute clusters.

The default taxonomic classification pipeline demonstrates the strengths of the workflow-based analysis approach, providing equal or even superior results than other established tools.

A high degree of scalability is another important aspect in order to cope with the increasing size of metagenome datasets; within MGX, this is achieved by means of a fast provisioning of novel, more efficient tools as well as the possibility to connect to multiple servers in parallel; this allows to distribute sequence data and computational workload across several sites while retaining the ability to compare results between servers.

As a part of the de.NBI initiative (German Network for Bioinformatics Infrastructure) funded by the German Federal Ministry for Education and Research, the BiGi service center for microbial genome research (comprising the Universities of Bielefeld and Gießen) currently maintains two MGX server instances, which distribute analysis jobs to compute clusters with over 9000 CPU cores (Bielefeld: 3350; Giessen: 6000), with additional resources such as ActiveMotif™ DeCypher^®;^ FPGA accelerator systems available to speed up sequence homology searches. For the future, we are planning to also provide portable virtual machine images or Docker containers to ease the deployment of an MGX server instance in for example cloud environments. Also, the collection of offered analysis workflows is constantly extended based on user demand and feedback from existing cooperation partners.

The modular design of the application greatly eases future enhancements such as the extension with novel visualization types or development of other additional components. In addition, a dedicated application programming interface (API) enables programmatic access and can be used to automate routine analysis tasks without large effort. With MGX, all analysis types can now be executed within one single framework, and all obtained results are stored in one single place, where they can easily be visualized and interpreted.

## Availability and requirements

Project name: MGXProject home page: https://mgx-metagenomics.github.io/Online documentation: https://mgx-metagenomics.github.io/guide/Operating system(s): Platform independentProgramming language: JavaLicense: GNU AGPLv3Artificial metagenomes generated in this study are published at ftp://ftp.cebitec.uni-bielefeld.de/pub/software/mgx/. For inquiries and general discussions, please contact mgx@Computational.Bio.Uni-Giessen.DE.

## Additional file

Additional file 1 Supplementary information. MGX supplemental figures and overview of analysis pipelines currently implemented within MGX. (PDF 355 kb)

## References

[1] Jansson JK, Prosser JI (2013). Microbiology: the life beneath our feet. Nature.

[2] Jaenicke S, Ander C, Bekel T, Bisdorf R, Dröge M, Gartemann KH, Jünemann S, Kaiser O, Krause L, Tille F (2011). Comparative and joint analysis of two metagenomic datasets from a biogas fermenter obtained by 454-pyrosequencing. PLoS ONE.

[3] Manichanh C, Rigottier-Gois L, Bonnaud E, Gloux K, Pelletier E, Frangeul L, Nalin R, Jarrin C, Chardon P, Marteau P (2006). Reduced diversity of faecal microbiota in Crohn’s disease revealed by a metagenomic approach. Gut.

[4] Sczyrba A, Hofmann P, Belmann P, Koslicki D, Janssen S, Droge J, Gregor I, Majda S, Fiedler J, Dahms E, Bremges A, Fritz A, Garrido-Oter R, Jorgensen TS, Shapiro N, Blood PD, Gurevich A, Bai Y, Turaev D, DeMaere MZ, Chikhi R, Nagarajan N, Quince C, Meyer F, Balvociute M, Hansen LH, Sorensen SJ, Chia BKH, Denis B, Froula JL, Wang Z, Egan R, Don Kang D, Cook JJ, Deltel C, Beckstette M, Lemaitre C, Peterlongo P, Rizk G, Lavenier D, Wu YW, Singer SW, Jain C, Strous M, Klingenberg H, Meinicke P, Barton MD, Lingner T, Lin HH, Liao YC, Silva GGZ, Cuevas DA, Edwards RA, Saha S, Piro VC, Renard BY, Pop M, Klenk HP, Goker M, Kyrpides NC, Woyke T, Vorholt JA, Schulze-Lefert P, Rubin EM, Darling AE, Rattei T, McHardy AC (2017). Critical assessment of metagenome interpretation—a benchmark of metagenomics software. Nat Methods.

[5] Goodwin S, McPherson JD, McCombie WR (2016). Coming of age: ten years of next-generation sequencing technologies. Nat Rev Genet.

[6] Mande SS, Mohammed MH, Ghosh TS (2012). Classification of metagenomic sequences: methods and challenges. Brief Bioinform.

[7] Sun S, Chen J, Li W, Altinatas I, Lin A, Peltier S, Stocks K, Allen EE, Ellisman M, Grethe J (2010). Community cyberinfrastructure for advanced microbial ecology research and analysis: the CAMERA resource. Nucleic Acids Res.

[8] Markowitz VM, Chen I-MA, Chu K, Szeto E, Palaniappan K, Grechkin Y, Ratner A, Jacob B, Pati A, Huntemann M (2012). IMG/M: the integrated metagenome data management and comparative analysis system. Nucleic Acids Res.

[9] Meyer F, Paarmann D, D’Souza M, Olson R, Glass EM, Kubal M, Paczian T, Rodriguez A, Stevens R, Wilke A (2008). The metagenomics RAST server—a public resource for the automatic phylogenetic and functional analysis of metagenomes. BMC Bioinformatics.

[10] Hunter S, Corbett M, Denise H, Fraser M, Gonzalez-Beltran A, Hunter C, Jones P, Leinonen R, McAnulla C, Maguire E (2014). EBI metagenomics-a new resource for the analysis and archiving of metagenomic data. Nucleic Acids Res.

[11] White JR, Arze C, Matalka M, et al.CloVR-Metagenomics: Functional and taxonomic microbial community characterization from metagenomic whole-genome shotgun (WGS) sequences—standard operating procedure, version 1.0. 2011. http://precedings.nature.com/documents/5886/version/3.

[12] Merchant N, Lyons E, Goff S, Vaughn M, Ware D, Micklos D, Antin P (2016). The iPlant collaborative: cyberinfrastructure for enabling data to discovery for the life sciences. PLoS Biol.

[13] Jiménez DJ, Dini-Andreote F, Ottoni JR, Oliveira VM, Elsas JD, Andreote FD (2015). Compositional profile of *α*/ *β*-hydrolase fold proteins in mangrove soil metagenomes: prevalence of epoxide hydrolases and haloalkane dehalogenases in oil-contaminated sites. Microb Biotechnol.

[14] Goecks J, Nekrutenko A, Taylor J (2010). Galaxy: a comprehensive approach for supporting accessible, reproducible, and transparent computational research in the life sciences. Genome Biol.

[15] Linke B, Giegerich R, Goesmann A (2011). Conveyor: a workflow engine for bioinformatic analyses. Bioinformatics.

[16] Altschul SF, Gish W, Miller W, Myers EW, Lipman DJ (1990). Basic local alignment search tool. J Mol Biol.

[17] Suzuki S, Kakuta M, Ishida T, Akiyama Y (2014). GHOSTX: an improved sequence homology search algorithm using a query suffix array and a database suffix array. PloS ONE.

[18] Buchfink B, Xie C, Huson DH (2015). Fast and sensitive protein alignment using DIAMOND. Nat Methods.

[19] R Core Team. R: a language and environment for statistical computing. Vienna, Austria: R Foundation for Statistical Computing; 2014. R Foundation for Statistical Computing. http://www.R-project.org/. Accessed 3 Sept 2017.

[20] Urbanek S, Hornik K, Leisch F, Zeileis A (2003). Rserve—a fast way to provide R functionality to applications. Proc. of the 3rd International Workshop on Distributed Statistical Computing (DSC 2003), ISSN 1609-395X.

[21] Wood D, Salzberg S (2014). Kraken: ultrafast metagenomic sequence classification using exact alignments. Genome Biol.

[22] Menzel P, Ng KL, Krogh A (2016). Fast and sensitive taxonomic classification for metagenomics with Kaiju. Nat Commun.

[23] Kim D, Song L, Breitwieser FP, Salzberg SL (2016). Centrifuge: rapid and sensitive classification of metagenomic sequences. Genome Res.

[24] Truong DT, Franzosa EA, Tickle TL, Scholz M, Weingart G, Pasolli E, Tett A, Huttenhower C, Segata N (2015). MetaPhlAn2 for enhanced metagenomic taxonomic profiling. Nat Methods.

[25] Eddy S. HMMER3: a new generation of sequence homology search software. 2010. http://hmmer.janelia.org. Accessed 3 Sept 2017.

[26] Bateman A, Coin L, Durbin R, Finn RD, Hollich V, Griffiths-Jones S, Khanna A, Marshall M, Moxon S, Sonnhammer EL (2004). The Pfam protein families database. Nucleic Acids Res.

[27] Haft DH, Selengut JD, Richter RA, Harkins D, Basu MK, Beck E (2013). TIGRFAMs and genome properties in 2013. Nucleic Acids Res.

[28] Powell S, Szklarczyk D, Trachana K, Roth A, Kuhn M, Muller J, Arnold R, Rattei T, Letunic I, Doerks T (2012). eggNOG v3.0: orthologous groups covering 1133 organisms at 41 different taxonomic ranges. Nucleic Acids Res.

[29] Pruitt KD, Tatusova T, Maglott DR (2006). NCBI reference sequences (RefSeq): a curated non-redundant sequence database of genomes, transcripts and proteins. Nucleic Acids Res.

[30] Chen I-MA, Markowitz VM, Chu K, Palaniappan K, Szeto E, Pillay M, Ratner A, Huang J, Andersen E, Huntemann M (2017). IMG/M: integrated genome and metagenome comparative data analysis system. Nucleic Acids Res.

