# Supplementary material for: Flexible metagenome analysis using the MGX framework
Source: Microbiome. 2018 Apr 24;6:76. doi: 10.1186/s40168-018-0460-1 (PMC5937802; doi:10.1186/s40168-018-0460-1)
Supplement: Supplementary file 1 — Supplementary information. MGX supplemental figures and overview of analysis pipelines currently implemented within MGX. (PDF 355 kb) [file 40168_2018_460_MOESM1_ESM.pdf]

# Flexible metagenome analysis using the MGX framework

## Supplementary information

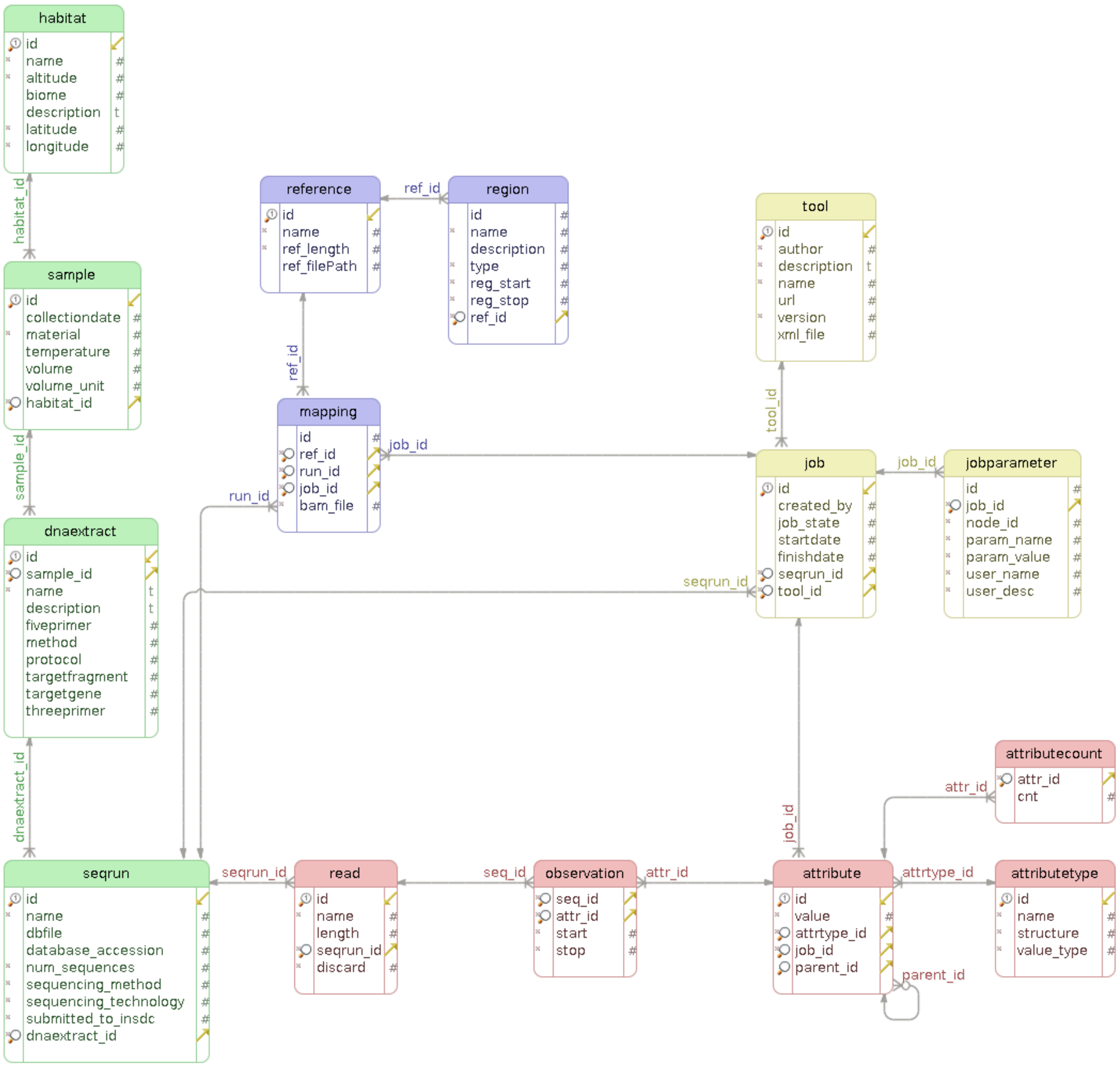


**Supplemental Figure 1: The MGX data model.** Sequence data and analysis results (red) in MGX are accompanied by corresponding metadata (green) describing a dataset's origin (habitat) and processing (sampling, DNA extraction, sequencing protocols) steps. Analysis jobs (yellow) are executed with customized parameters to annotate sequences with attributes or to perform alignment to known reference genomes (purple).

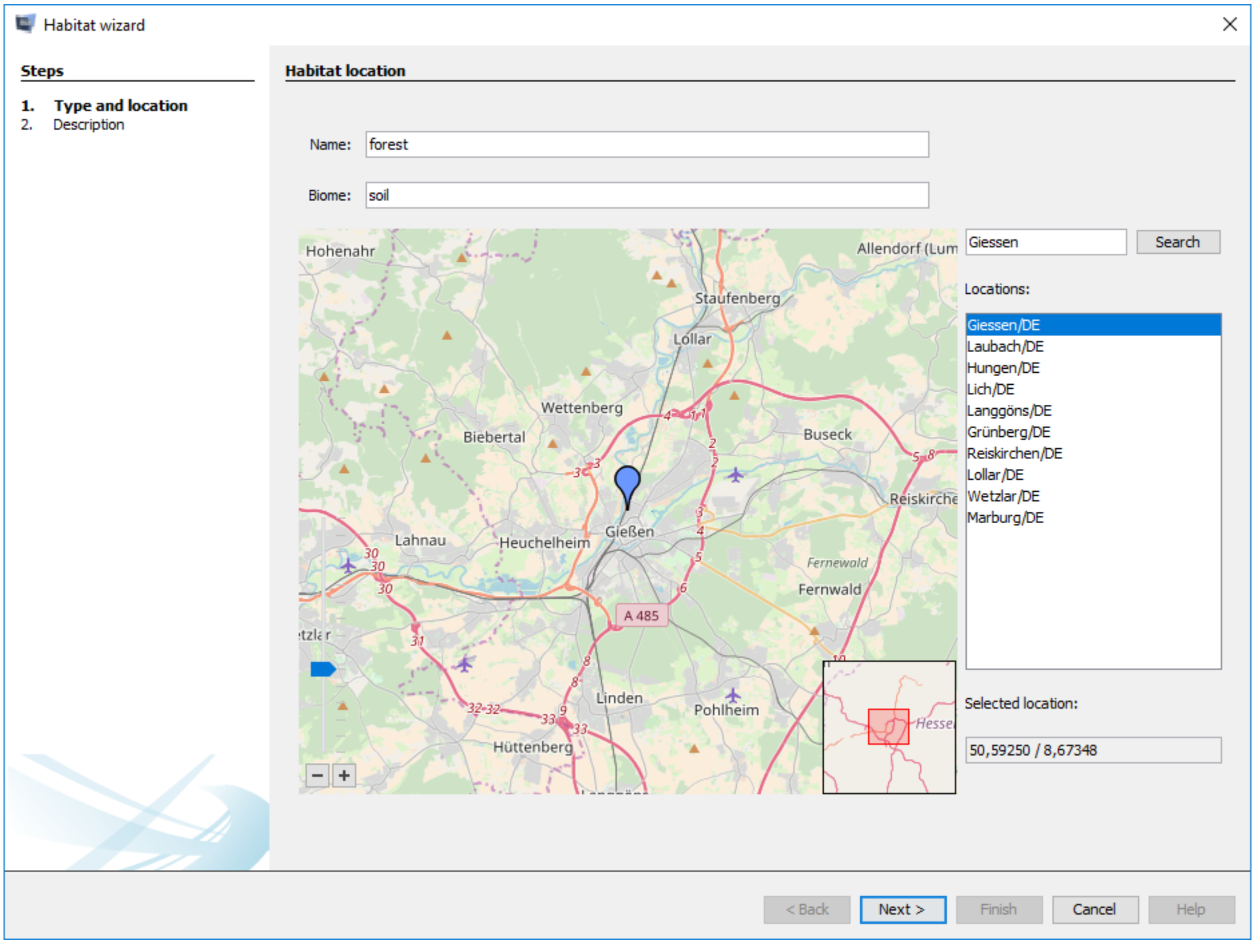


**Supplemental Figure 2: Metadata acquisition.** During initial data import, MGX provides user-friendly wizards allowing to enter and validate metadata which is stored with each dataset. The figure depicts the habitat wizard which is used to capture the geographical origin and biome type.

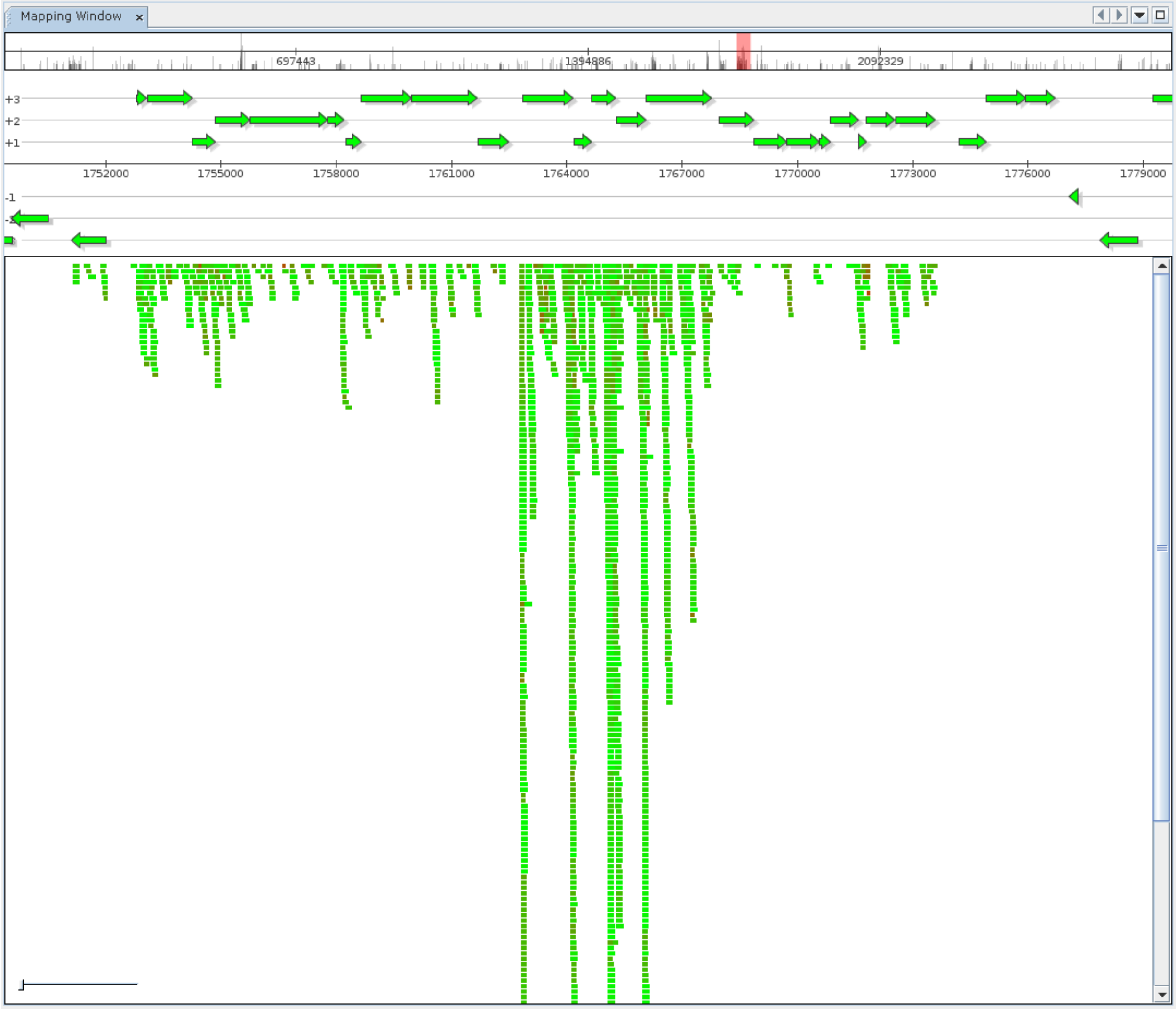


**Supplemental Figure 3: Reference mapping.** The reference mapping component showing alignment results for a metatranscriptome dataset mapped to the reference genome of one of the dominant organisms. From top to bottom, the component displays a) navigation and coverage histogram, b) currently selected interval and c) aligned DNA sequences for the interval. Color coding refers to relative sequence identity.

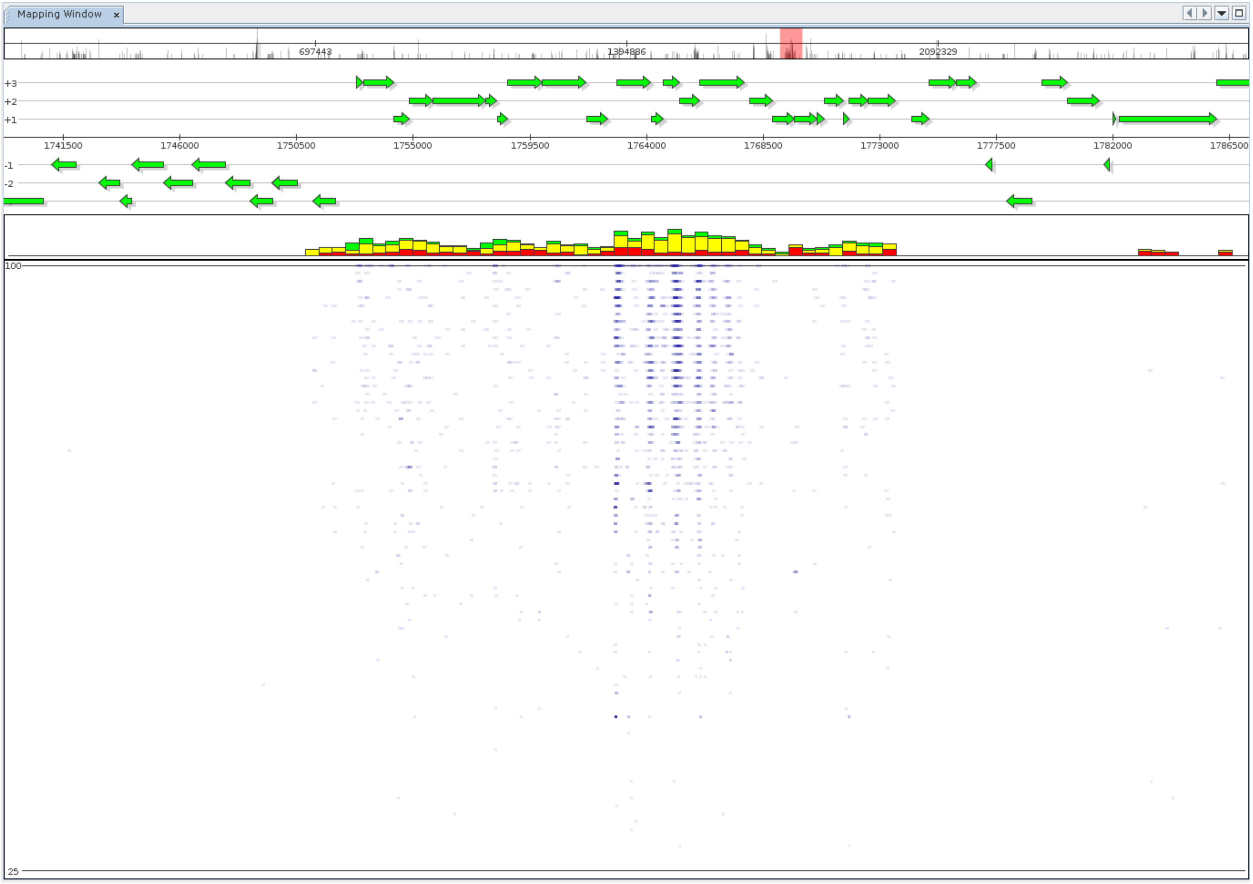


**Supplemental Figure 4: Fragment recruitment.** An alternate visualization mode is the generation of fragment recruitment plots, here showing the same data as described previously (Supplemental Figure 3). This view mode features the fragment recruitment plot itself and additionally provides stacked bars summarizing mapping identity within reference intervals, grouped into low (red), medium (yellow, $\geq 75\%$) and high (green, $\geq 97\%$) quality mappings.

| Name | Approach/Description |
|---|---|
| **Quality control** | |
| Read length | Distribution of sequence length |
| GC content | Distribution of GC content |
| **Taxonomic assignment** | |
| MetaCV[1] | Composition-based classification |
| Kraken[2] | k-mer based taxonomic assignment |
| RDP[3] | 16S rRNA gene fragment classification |
| Lowest-common-Ancestor (LCA) | LCA taxonomic classification compatible to MEGAN[4] |
| MetaPhyler[5] | Marker-based assignment |
| MetaPhlAn[6] | Marker-based assignment |
| MetaPhlAn 2[7] | Marker-based assignment |
| Kaiju[8] | Taxonomic assignment based on maximum exact matches |
| Centrifuge[9] | Rapid and sensitive classification of metagenomic sequences |
| **Functional profiling** | |
| EggNOG[10] | Assignment to COG groups and categories |
| KEGG[11] pathways | Assignment to KEGG pathway maps based on EC numbers |
| Pfam[12] | Identification of protein families in the Pfam database |
| TIGRFAMs[13] | Identification of protein families in the TIGRFAMs database |
| ClusterMine360[14] | Screening for PKS/NRPS domains |
| dbCAN[15] | Identification of carbohydrate-active enzymes |
| FunGene[16] | Functional analysis based on the FunGene functional gene repository |
| **Antimicrobial resistance** | |
| MVirDB[17] | Identification of antibiotic resistance genes, toxins or virulence factors |
| ARDB | Antibiotic resistance gene screening based on the ARDB database |
| BacMet | Antibacterial biocide- and metal-resistance gene annotation |
| CARD | Resistance gene annotation using the CARD database |
| ARG-ANNOT | Antibiotic Resistance Gene-ANNOTation |
| **Reference mapping** | |
| Bowtie[18] | Alignment to reference genomes based on the Bowtie2 aligner |
| FR-HIT[19] | Fragment recruitment employing FR-HIT |
| Magic-BLAST | Fragment recruitment |
| **Amplicon analysis** | |
| mothur[20] | 16S rRNA classification |
| RDP[3] | 16S rRNA and ITS amplicon classification |
| Qiime[21] | 16S rRNA amplicon classification |
| **Templates** | |
| BestHit-Blast | Annotation of best Blast hit employing a user-provided database |
| BestHit-HMM | Annotation of best HMMer hit employing user-provided HMM models |

**Supplemental Table 1:** Overview of analysis pipelines currently implemented within MGX.

## References


1. Liu, J. *et al.* Composition-based classification of short metagenomic sequences elucidates the landscapes of taxonomic and functional enrichment of microorganisms. *Nucleic Acids Research* **41**, gks828 (2012).

2. Wood, D. & Salzberg, S. Kraken: ultrafast metagenomic sequence classification using exact alignments. *Genome Biology* **15**, R46 (2014).

3. Wang, Q., Garrity, G. M., Tiedje, J. M. & Cole, J. R. Naïve bayesian classifier for rapid assignment of rRNA sequences into the new bacterial taxonomy. *Applied and Environmental Microbiology* **73**, 5261–5267 (2007).

4. Huson, D. H., Mitra, S., Ruscheweyh, H.-J., Weber, N. & Schuster, S. C. Integrative analysis of environmental sequences using MEGAN4. *Genome Research* **21**, 1552–1560 (2011).

5. Liu, B., Gibbons, T., Ghodsi, M., Treangen, T. & Pop, M. Accurate and fast estimation of taxonomic profiles from metagenomic shotgun sequences. *BMC Genomics* **12**, S4 (2011).

6. Segata, N. *et al.* Metagenomic microbial community profiling using unique clade-specific marker genes. *Nature Methods* **9**, 811–814 (2012).

7. Truong, D. T. *et al.* MetaPhlAn2 for enhanced metagenomic taxonomic profiling. *Nature Methods* **12**, 902–903 (2015).

8. Menzel, P., Ng, K. L. & Krogh, A. Fast and sensitive taxonomic classification for metagenomics with Kaiju. *Nature Communications* **7**, 11257–11257 (2016).

9. Kim, D., Song, L., Breitwieser, F. P. & Salzberg, S. L. Centrifuge: rapid and sensitive classification of metagenomic sequences. *Genome Research* **26**, 1721–1729 (2016).

10. Powell, S. *et al.* eggNOG v3.0: orthologous groups covering 1133 organisms at 41 different taxonomic ranges. *Nucleic Acids Research* **40**, D284–D289 (2012).

11. Kanehisa, M. *et al.* Data, information, knowledge and principle: back to metabolism in KEGG. *Nucleic Acids Research* **42**, D199–D205 (2014).

12. Bateman, A. *et al.* The Pfam protein families database. *Nucleic Acids Research* **32**, D138–D141 (2004).

13. Haft, D. H. *et al.* TIGRFAMs and genome properties in 2013. *Nucleic Acids Research* **41**, D387–D395 (2013).

14. Conway, K. R. & Boddy, C. N. ClusterMine360: a database of microbial PKS/NRPS biosynthesis. *Nucleic Acids Research* **41**, D402–D407 (2013).

15. Yin, Y. *et al.* dbCAN: a web resource for automated carbohydrate-active enzyme annotation. *Nucleic Acids Research* **40**, W445–W451 (2012).

16. Fish, J. A. *et al.* FunGene: the functional gene pipeline and repository. *Frontiers in Microbiology* **4**, 291 (2013).

17. Zhou, C. *et al.* MvirDB-a microbial database of protein toxins, virulence factors and antibiotic resistance genes for bio-defence applications. *Nucleic Acids Research* **35**, D391–D394 (2007).

18. Langmead, B. & Salzberg, S. L. Fast gapped-read alignment with Bowtie 2. *Nature Methods* **9**, 357–359 (2012).

19. Niu, B., Zhu, Z., Fu, L., Wu, S. & Li, W. FR-HIT, a very fast program to recruit metagenomic reads to homologous reference genomes. *Bioinformatics* **27**, 1704–1705 (2011).

20. Schloss, P. D. *et al.* Introducing mothur: open-source, platform-independent, community-supported software for describing and comparing microbial communities. *Applied and Environmental Microbiology* **75**, 7537–7541 (2009).

21. Caporaso, J. G. *et al.* QIIME allows analysis of high-throughput community sequencing data. *Nature Methods* **7**, 335–336 (2010).
